# Social Network Interventions for HIV Transmission Elimination

**DOI:** 10.1007/s11904-020-00524-z

**Published:** 2020-07-28

**Authors:** Jade Pagkas-Bather, Lindsay E. Young, Yen-Tyng Chen, John A. Schneider

**Affiliations:** 1 Department of Medicine, University of Chicago, 5841 South Maryland Avenue, MC 5065, Chicago, IL 60637 USA; 2 Chicago Center for HIV Elimination, 5841 South Maryland Avenue, MC 5065, Chicago, IL 60637 USA; 3 Department of Public Health Sciences, University of Chicago, Chicago, IL 60637 USA

**Keywords:** HIV, PrEP, Networks, Intervention, Prevention, Sex, MSM

## Abstract

**Purpose of Review:**

Network interventions for HIV prevention represent a potential area for growth in a globalizing world, where persons are more easily connected to one another through social media and networking applications. The basic tenets of network interventions such as (1) selection of a change agent, (2) segmentation, (3) induction, and (4) alteration represent myriad ways to structure network interventions for HIV prevention with the potential for large public health impact.

**Recent Findings:**

Recent studies have employed the use of social networking websites such as Facebook to identify key persons to recruit others and disseminate information aimed at decreasing HIV transmission and improving safe sex practices among groups who are more vulnerable to HIV acquisition. Many of these interventions have successfully decreased HIV risk behaviors as well as decreased the spread of HIV among intervention cohorts.

**Summary:**

Network interventions for HIV prevention provide more opportunities to reach populations who have not been reached through typical efforts employed in clinical and public health settings, though they are not currently widely employed by the public health community and other stakeholders.

## Introduction

Social networks as a unit of social structure represent an essential feature of public health and an opportunity for interventionists to engage networks around HIV prevention through network characterization, visualization, analysis, and intervention. The goal of network analysis is to explain the behavior of groups of individuals within their context and the systems and interrelationships that exist as a result of the connections between said groups [1]. Relationships influence a person’s behavior above and beyond the influence of individual attributes [2]. This is particularly true of health behaviors—smoking, eating, and sex are all socially shaped behaviors [3–5]. Diffusion of information and innovation often occurs through personal networks, which are shaped by geographic, ethnic, and socioeconomic factors [6, 7]. In resource-rich nations, there is evidence that social network factors are critical to the spread of HIV, but most of this research has focused on social relationships instead of probing the specific characteristics of social networks that mediate these outcomes, such as HIV prevalence, concurrency, size of network, and sexual behavior [8–10]. Reaching a vulnerable population targeted for HIV prevention is not straightforward because such populations do not acquire information about new strategies from public media; instead, these individuals often obtain and transmit information primarily through their informal social networks, comprised mainly of friends or other peers [11]. Although social network analysis has examined the relationship between social networks and health since the 1970s, actual social network interventions are limited [12].

The majority of network interventions for health improvement, especially as pertains to disease prevention, provide methodologies for implementing behavioral change strategies that include a role for social influence, norms and peer influences [13]. In this way, network science rests within the spectrum of implementation science which aims to bridge gaps between knowledge and behavior, better known as the “know-do gap,” often imperative to bringing public health initiatives of any kind to scale [14]. An implementation science approach can take on some of the features of a network intervention. Specifically, implementation science can inform the adaptive potential of network interventions, which can be tailored to successful use of a biomedical intervention, such as remaining virally suppressed for HIV-positive persons or HIV-negative for persons using PrEP for HIV prevention [15]. Network interventions for HIV prevention like much of implementation science aims to bridge the gap between utility, effectiveness, and application [16].

A central tenet of network interventions is the notion that behavior change is enacted through explicit or implicit mechanisms of influence among individuals that are close to one another [6]. The intervention can itself be selection of the change agent, relying on a single individual or groups of individuals to promote the intervention and keep the message constant. In this sense, the messenger can often be more important than the message. One might consider that messages promoted by public health officials, circumcision for example, may be of limited interest to others, even those at increased risk for HIV [17]. In fact a common when messages are of limited interest, the processing strategy tends to focus more on the messenger [18]. Further, as uncertainty increases in a particular context or message, the reliance on more transparent messenger characteristics, such as obvious status signals, (markers of prestige) heightens in importance [19, 20].

Research on the diffusion of health behaviors within the context of network interventions has been active in areas such as fertility preferences [21] and pharmaceutical marketing [22], but much less so for the diffusion of HIV prevention [23•]. Typically, diffusion in the context of HIV prevention has been conceptualized as the dissemination of effective behavioral interventions to community-based organizations and local health departments such as the effective distribution of condoms within bathhouses to reduce HIV and other STI transmission [24, 25], with less focus on the selection of change agents to help facilitate behavior modifications. Because until recently there have been few HIV prevention innovations using antiretrovirals such as PrEP, the power of HIV innovation diffusion models based in social network structures have not been explored. Determining thresholds for the diffusion of biomedical (PrEP) and other HIV prevention innovations will bring us closer to determining which individuals and groups these interventions are likely to reach and benefit, and will inform strategies for accelerating adoption of these novel therapies within complex environments, such as a sexual and social networks, which often overlap [26]. Given the many obstacles to behavior change, there is value in understanding the prevention potential for antiretroviral-based therapies when there may be only minimal behavior change, such as condom use for HIV prevention [27].

## A Taxonomy for Network Intervention

In his seminal work, Thomas Valente describes four typologies of network interventions [28]. These four network intervention typologies include change agent (Type I), segmentation (Type II), induction (Type III), and alteration (Type IV) (Table 1). In this review, we use this organizing structure and describe how these interventions have or could be applied to HIV transmission elimination as well as future directions for research.

**Table 1:** Four types of network interventions

Intervention typology	Key players	Examples	Studies employing network intervention	Future directions
Type I. Change agent	Popular opinion leaders (POL)—those selected by a group as influential within a network context	Use of participants with large social networks to recruit participants	Young et al., 2018	Better and more precise identification of POL in network interventions to galvanize others for change through use of social media or stakeholder interviewing
		Popular gay men were recruited to provide HIV education resulting in decreased unprotected anal intercourse	Kelly et al., 1997	
		Training Black MSM POL reduced unprotected anal intercourse	Jones et al., 2008	
		Participants linked to POL vs. AIDS education group and both groups had decreased HIV risk behavior	NIMH, 2010	
Type II. Segmentation	Group level interventions which gain in popularity among individuals such as Instagram, Facebook, and Twitter	Participants linked to PrEP peer leaders	Patel et al., 2018	Development of various communication strategies for addressing different groups for the same intervention
		Trained house ball leaders to disseminate safe sex information leading to decreased HIV risk behaviors	Hosek et al., 2015	
Type III. Induction	Word of mouth interventions that impact the spread of behavior—the process by which an internet article “goes viral”	HIV+ persons recruited within their networks to increase testing	Kimbrough et al., 2009	Increased utilization of respondent driven sampling in studies and network interventions
Type IV. Alteration	Identification of social supports among HIV-positive women in order to increase membership within the network	HIV+ women encouraged to expand their support network through dyadic relationships	Wingood et al., 2004	Linking HIV positive persons to support groups based on pre-identified social needs

## Peer Change Agent Interventions—Type I

Perhaps the most classic and intuitive network intervention is the peer change agent intervention. Peer change agent interventions identify influential individuals within a target population and train them to champion a desired behavior within their networks. The act of championing a behavior can take many forms—e.g., educating peers about the benefits of a behavior, modeling the behavior itself, and providing support to peers who are in the process of adopting the behavior—and has been applied to encourage HIV-related behavioral or attitudinal changes in dyadic [29], personal network [30], or community contexts [31].

Although change agents can be chosen on the basis of a variety of salient characteristics including their desire to help, aspects of their personality (e.g., leadership, innovativeness), or their experience with the desired behavior, what grants a peer change agent their credibility rests largely in their social standing in organic social networks among members of the target population [32, 33]. Thus, most peer change agent interventions tend to select candidate change agents on the basis of peer nominations or their actual structural position vis-à-vis other peers in an observed social network [28].

In the context of HIV prevention, the Type I change agent interventions are the most commonly utilized and reported on with documented efficacy in rigorously conducted studies. The prototypical peer change agent intervention is the Popular Opinion Leader (POL) model [34], which draws on peer nominations to identify well-liked individuals or “natural leaders” in a targeted population. POL interventions have been found to be effective for promoting protective behaviors like condom use and other risk reduction practices. For example, in a POL intervention for Black men who have sex with men (MSM) in three southern US cities, researchers reported decreases in episodes of condomless anal sex and increases in condom use for insertive and receptive anal intercourse at 8 and 12 months [35]. Similarly, another POL study engaging MSM who frequented gay bars in New York, Washington, West Virginia, and Wisconsin reported decreases in condomless sex [36].

That said, some POL studies have shown more mixed results. In a randomized trial across five countries, the deployment of “natural leaders” led to a 33% reduction in HIV risk behaviors among individuals assigned to the intervention. However, these reductions were experienced mostly among those at highest-risk (e.g., with an STI engaging in high risk sexual activities) rather than the general population [37]. Taken together, these findings suggest that there is some benefit to information dissemination that is both of and for the network, provided the community for whom the intervention is intended is well defined and circumscribed. Future research continues to require randomized or other rigorously conducted community engaged experiments to avoid social desirability bias and other measurement problems that faced earlier studies.

The POL model may also be hampered by its over-reliance on change agents who are most “popular” in a network, a position that often requires an individual to maintain conformity to the status quo rather than change it [38]. This has led to suggestions that individuals who are “network bridges” may be better suited as change agents. Bridging actors are those who connect otherwise disconnected groups in a social environment. As such, they are well positioned to accelerate the spread of new ideas and behaviors across loosely or non-connected regions of a network or community [6]. Additionally, compared with “popular” actors, bridging actors may be more receptive to behavior change as they have less pressure to support prevailing norms and behaviors [39] and are less concerned about incurring a reputation cost for new and potentially disapproved behavior [40]. For these reasons, occupying a bridging position may be indicative of being open to new ideas and practices [41, 42] and being willing to share them with others.

Although the intentional use of network bridges remains an underutilized peer change agent intervention strategy (see Young et al., 2018 for an exception), several studies reveal important clues about their potential efficacy for promoting HIV prevention. In a study involving Indian MSM [43], candidate peer change agents who were also network bridges demonstrated greater innovativeness than their more centrally located (i.e., popular) counterparts, suggesting a greater openness to communicating about novel practices. And, in two studies exploring social network factors associated with peer leader performance, individuals in bridging positions reported having more HIV-related conversations with peers [43] and were more effective at recruiting their peers to serve as peer change agents [33].

## Segmentation Interventions—Type II

In contrast to interventions that employ individual change agents as the proponents of behavior change, segmentation interventions identify groups of people to change at the same time [28]. Groups can be defined *a priori* as is the case when an innovation is introduced into distinct communities of practice like House Ball Community (HBC) [44], which is a prominent feature in African American and Latinx LGBTQ communities, or in mutually exclusive personal networks.

For example, Hosek et al. (2015) tailored an evidence-based intervention for use within the HBC called Promoting Ovahness through Safer Sex Education (POSSE); the intervention recruited and trained leaders from within the HBC to deliver risk reduction messages to their peers, resulting in statistically significant declines in self-reported partner concurrency, condomless anal intercourse, and intercourse with partners with unknown HIV status [45]. And, in their ongoing peer-based social network intervention Empowering with PrEP (E-PrEP), Patel et al. (2018) use a cluster-randomized design to keep study participants clustered with the PrEP peer leaders who recruited them into the study. In doing so, they approximate real-world diffusion circumstances and minimize contamination within peer networks, which is always a challenge when intervening in a known community with preexisting network ties [46]. Results of this study are forthcoming.

Group segmentation can also be based on a particular attribute of the individuals in the target population. A promising terrain in which to employ attribute-based segmentation in the service of HIV prevention and care engagement is the “hook-up” or dating application (app). “Hook-up” apps for MSM (e.g., Grindr) allow users to signal their partner sero-sorting preferences in their profiles. An intervention that clusters these sero-sorters on the basis of their negative or positive HIV status would allow researchers to test the effectiveness of status-neutral approaches to testing and treatment engagement in both behavioral communities [47]. Given that many studies show MSM who use dating or hook-up apps to meet sexual partners have higher rates of STI, group segmentation in collaboration with these geosocial networking apps represents an area for network interventions that have not yet been widely explored [48, 49].

Finally, unlike the previous segmentation strategies that draw on preexisting categorical distinctions to define groups, segmentation can also be artificially imposed on a target population in the service of an intervention that aims to encourage group-based interactions and social learning. The HOPE intervention study implemented in Peru and later in Los Angeles used this segmentation approach toward promoting HIV testing among MSM, and is evidence of the effectiveness of a Type II network intervention as an HIV prevention intervention. Specifically, MSM participants were assigned to secret Facebook groups with designated peer leaders who were trained to encourage group members to test for HIV, which resulted in greater numbers of intervention group members testing for HIV and using condoms [50, 51]. The key to segmentation is the need to match leaders to subgroups within a network in order to have the greatest impact.

## Network Mobilization Interventions—Type III

Network mobilization or network induction is an intervention strategy that stimulates peer-to-peer interaction to create information cascades or behavioral diffusion through existing social pathways among network members [28]. The Type III intervention represents a second class of network interventions that have been found to be effective in HIV prevention (i.e., CDC’s EBI—Social Network Strategy). Although not an intervention strategy, peer-based sampling techniques like respondent-driven sampling (RDS) and snowball sampling are examples of how these induced information cascades have been applied to study recruitment for HIV prevention studies.

An example of how induction works as both a recruitment and intervention strategy is next-generation social network strategies (SNS) for HIV testing [52, 53]. Past research has shown that individuals who are members of the same social network are more likely to have similar HIV risk potential [53]. As a result, testing programs that include recruitment of social contacts in addition to risk contacts have recently been promoted by the CDC as an effective HIV testing intervention [52, 53]. This strategy identifies HIV-positive individuals and individuals who are at risk for acquiring HIV and asks them to recruit persons from their social network for testing in exchange for an incentive. In so doing, the reach of the testing program increases as does the volume of testers overall. Data show that a proportion of those tested through SNS are newly identified HIV infections, which is routinely higher than the prevalence found via publicly funded counseling, testing, and referral sites [54] and as such is an effective Type III network intervention.

Building upon classical partner services and the Social Network Strategy, the Transmission Reduction Intervention Project (TRIP) conducted network-based recruiting, counseling, and testing in Odessa, Ukraine; Athens, Greece; and Chicago, Illinois. TRIP’s primary goal was to increase early detection of recently HIV-infected individuals [55]. A secondary goal, at the Chicago site only, was to increase the ability to locate individuals with active syphilis infection [55]. A two-step approach (a person who recently seroconverted was followed for two rounds of the study in order to capture additional HIV-positive contacts) was utilized where network chain recruitment started with “seeds”—either recently or long-term HIV-positive—and continued no further than two steps from a person living with HIV, repeating the process if another person living with HIV was identified at either the first or second step in the process [55]. TRIP was found to be effective at yielding individuals newly diagnosed with HIV, including recent HIV infection and active syphilis infection [55, 56], the first step in any care continuum intervention. The two-step approach was utilized in order to remain within each individual’s risk network environment; anything beyond this was considered at the time to be outside the immediate risk network. However, future work to determine whether greater chain length can yield more new diagnoses is worth considering. Further, determining the optimal network mobilization strategy following molecular cluster identification is also a growing area of interest and investigation [57, 58].

Inducing HIV prevention information cascades can also be achieved in nonclinical, naturalized social environments. Social networking platforms like Facebook, Twitter, Instagram, and YouTube enable rapid content sharing and electronic Word-of-Mouth (eWOM) cascades that can be leveraged for health promotion campaigns [59]. The *#TruvadaWhore* social media campaign exemplifies what virality can do for HIV prevention awareness and advocacy. Launched in response to the way in which PrEP users in the gay community were being stigmatized as promiscuous “Truvada Whores” [60], the *#TruvadaWhore* campaign defiantly reappropriated this “slut shaming” to be worn instead as a badge of pride. On Twitter alone, the use of the Truvada Whore hashtag resulted in thousands of tweets that brought attention to PrEP as a viable HIV prevention innovation that strove to destigmatize its use and that generated political activism. Grassroots campaigns like this can offer lessons for interventionists who want to generate interest and enthusiasm for different prevention modalities through peer-to-peer messaging.

## Alteration Interventions—Type IV

Changing the network is likely the most difficult network intervention to implement and potentially the strategy with the most ethical challenges, whereby networks and social spheres are manipulated for the potential to illicit behavior change. This is not unlike the ways in which Facebook or Grindr may suggest particular “friends” or potential partners for users on their platforms [61]. Interventions that aim to augment network structure seek to do one of three things as follows: (i) add/delete nodes, (ii) add/delete links, or (iii) rewire existing links [28]. Alteration network intervention strategies in public health have yet to be fully realized and there is the least evidence available for Type IV interventions in the HIV prevention space. Adding nodes is a long-standing approach to health behavior change with external agents like community health workers being deployed in communities to accelerate the pace of change. A study conducted by Wingood et al. (2004) relied on HIV-positive women to identify supportive members of their communities to expand the network of HIV-positive individuals by emphasizing gender pride and encouraging women to seek out dyadic relationships to increase the number of persons within their support network, resulting in decreased bacterial STI acquisition and risky sexual behaviors such as condomless intercourse [62]. Few interventions within the HIV prevention arena have sought to increase numbers of persons within a particular network, though HIV prevention peer navigators have been used to encourage persons to engage in HIV prevention behaviors such as taking and adhering to oral PrEP [63]. Within public health contexts, it is likely that influencing ties by strengthening connections or by introducing previously unconnected network members who have a common need (e.g., a buddy system) could be other appropriate uses of this approach [64–66].

## Discussion

The decision to use network interventions within the HIV prevention cascade involves a complex set of features aimed at targeting multiple persons for maximum public health impact. Clearly, implementation science can contribute to network intervention effectiveness, though how and when we utilize said interventions depends largely on inner and outer contexts, and the desired outcome, in addition to the desired scope of prevention efforts and ability to “scale-out” these interventions [65, 66]. For example, implementation of a change agent may be employed when the intervention aims to target a smaller subset of persons with similar social and sexual networks [43], or that are within a particularly segregated community area such as the South Side of Chicago or West Louisville, Kentucky. The limitations of this approach would be potential recall bias, thus limiting the breadth and reach of the network intervention as it relies on the individual to relay the scope of their social or sexual network, thus leading to incomplete network characterization [67], as in a study involving the sexual partners of Indian truck drivers [42]. One way to overcome recall bias is to use network data itself, as in *PrEP Chicago* where digital network data from Facebook was used to provide a more expansive definition of a person’s social network [23•]. An additional limitation is that a change agent is only influential to those who follow them, meaning that their reach is circumscribed solely within the context of their particular network, excluding persons who are outside of their sphere of influence.

Larger scale HIV prevention interventions can be organized through the process of segmentation, which can take on geographic, sociodemographic, psychographic, and sociometric characteristics in order to reach a subset of the population at specific risk for HIV [12]. The utilization of social media for network interventions has increased in popularity given the potential to reach large groups of individuals stratified by interest, location, and demographics. Utilization of Facebook, Instagram, and mobile social networking applications geared toward both social and sexual connectivity (e.g., Grindr, JACK’D)[68] have allowed researchers to set their work within the context of highly utilized technology to reach large groups of individuals [69]. In a study by Broaddus et al. 2015, Black MSM who used social media were more likely to engage in sex with HIV transmission potential than those who did not [70]. Thus, utilization of social media to enhance network interventions may provide opportunities to impact groups vulnerable to HIV. The difficulty of segmentation may lie in identifying key players in delivery of HIV prevention messages within the broader context of social media utilization [12].

The process of social network recruitment is rooted in peer-based recruitment, and can serve to identify populations at risk for HIV who are less likely to interface with healthcare settings due to discrimination or isolation, such as young Black MSM in the United States [71, 72]. The chain referral system generated by the induction approach to network interventions can broaden the reach of HIV prevention efforts by calling on an initially selected group of persons who then increase the diversity of the referrals over time as the chain-generated network increases in size [72]. An unavoidable limitation to this approach is that it depends on patterns of contact identified by the public health interventionist—i.e., men having sex with men, which make it less generalizable to a larger cohort of individuals [72]. Induction as a network intervention proves most useful when examining the broader characteristics of a key population of interest.

Alteration may be the most difficult network intervention to characterize within the context of HIV prevention efforts as it is less well described and utilized. Alteration as an HIV prevention strategy represents an area for further development within the context of network interventions. Though given its potential for desired outcomes in HIV-positive persons, there is potential to develop studies to test its feasibility within the HIV prevention cascade.

## Conclusion

Network interventions represent a growing area for HIV prevention efforts surrounding PrEP and other biomedical and behavioral interventions aimed at stemming new infections. Understanding groups who are most vulnerable to HIV infection, change makers within their social spheres, and methodologies for increasing both education and thereby, decision-making capacity may broaden the success of public health interventions to reduce HIV transmission. Implementation science becomes increasingly important if network interventions are to be successful, particularly with close attention paid to (1) stakeholder buy-in and context given that network data collection can be extensive, sensitive, and (2) that most public health HIV researchers and service providers have been dominated by psychological underpinnings of prevention and focused primarily at the individual level. Understanding the networks therein may allow for more successful outcomes for HIV prevention by emphasizing the influence of social systems and the environment on behaviors related to sexual health.
